# Development and evaluation of a rapid molecular diagnostic test for Zika virus infection by reverse transcription loop-mediated isothermal amplification

**DOI:** 10.1038/s41598-017-13836-9

**Published:** 2017-10-18

**Authors:** Yohei Kurosaki, Danyelly Bruneska Gondim Martins, Mayuko Kimura, Andriu dos Santos Catena, Maria Amélia Carlos Souto Maior Borba, Sandra da Silva Mattos, Haruka Abe, Rokusuke Yoshikawa, José Luiz de Lima Filho, Jiro Yasuda

**Affiliations:** 1 Institute of Tropical Medicine (NEKKEN), Nagasaki University, Nagasaki, 852-8523 Japan; 2 Laboratory of Immunopathology Keizo Asami (LIKA), Federal University of Pernambuco (UFPE), Recife, 50670-901 Brazil; 3 Graduate School of Biomedical Sciences and Program for Nurturing Global Leaders in Tropical and Emerging Communicable Diseases, Nagasaki University, Nagasaki, 852-8523 Japan

## Abstract

The recent outbreak of Zika virus (ZIKV) disease caused an enormous number of infections in Central and South America, and the unusual increase in the number of infants born with microcephaly associated with ZIKV infection aroused global concern. Here, we developed a reverse transcription loop-mediated isothermal amplification (RT-LAMP) assay using a portable device for the detection of ZIKV. The assay specifically detected ZIKV strains of both Asian and African genotypes without cross-reactivity with other arboviruses, including Dengue and Chikungunya viruses. The assay detected viral RNA at 14.5 TCID_50_/mL in virus-spiked serum or urine samples within 15 min, although it was slightly less sensitive than reference real time RT-PCR assay. We then evaluated the utility of this assay as a molecular diagnostic test using 90 plasma or serum samples and 99 urine samples collected from 120 suspected cases of arbovirus infection in the states of Paraíba and Pernambuco, Brazil in 2016. The results of this assay were consistent with those of the reference RT-PCR test. This portable RT-LAMP assay was highly specific for ZIKV, and enable rapid diagnosis of the virus infection. Our results provide new insights into ZIKV molecular diagnostics and may improve preparedness for future outbreaks.

## Introduction

Zika virus (ZIKV) was first identified in Uganda in 1947. Since then, human infections were found across Africa and Southeast Asia^1,2^. In May 2015, the first case of infection with ZIKV on the South American continent was reported in Brazil^2,3^. By early 2016, the number of ZIKV infections in Brazil increased dramatically, and an unusual number of cases of foetal and newborn microcephaly associated with ZIKV infection in pregnant women were reported^4,5^. To control ZIKV disease outbreaks and the spread of ZIKV infections, the World Health Organization declared a Public Health Emergency of International Concern in February 2016. ZIKV infections have also been reported in other American countries and continue to expand.

A particular concern with respect to ZIKV infection is the increased risk of congenital central nerve system malformations, including microcephaly as well as arthrogryposis and spontaneous abortion caused by maternal infection during the first or second trimester of pregnancy^6,7^. In addition, sexual transmission and the probable transmission through blood transfusions have been reported^8–10^. ZIKV infection usually causes a mild and self-limiting illness, e.g., fever, rash, arthralgia, and conjunctivitis. Since these clinical symptoms are commonly observed in infections with other arboviruses, such as Dengue (DENV) and Chikungunya (CHIKV) viruses, it is difficult to diagnose ZIKV infection by clinical symptoms alone^1,2^. Therefore, accurate laboratory diagnosis to identify ZIKV infections is urgently required, especially for pregnant women who are at risk of bearing children with microcephaly.

ZIKV is a positive-stranded RNA virus belonging to the genus *Flavivirus* in the family *Flaviviridae*. ZIKV shares its vector, the *Aedes* mosquito, with other flaviviruses, including DENV, Yellow fever virus (YFV), and CHIKV^1^. ZIKV has been isolated from humans in East and West Africa and in Southeast Asia and Polynesian countries where the host mosquitoes, *A. aegypti* and *A. albopictus*, are found^1,11^. Based on phylogenetic analyses, these isolates can be categorised into two genotypes, African and Asian. Epidemiological studies have revealed that the recent outbreak of ZIKV in Brazil occurred via the introduction of a virus from French Polynesia, where an outbreak of the disease occurred in 2013^12^. All of the viruses isolated in Brazil and other countries on the American continent belong to the Asian genotype^13^.

In patients with ZIKV infection, the virus can be detected in several sample types, including blood, urine, saliva, and other body fluids^14–18^. The viral load in blood reaches a peak at 2 to 5 days after the onset of illness, but decreases rapidly thereafter. Therefore, it is difficult to detect ZIKV in blood samples from patients after the acute phase of infection, even with sensitive molecular diagnostic methods, such as reverse transcription-polymerase chain reaction (RT-PCR)^14,18,19^. The virus can be detected in urine samples for longer durations (>7–14 days after the onset of symptoms) than in those for blood samples^14^. Currently, blood and urine samples are typically used for the molecular diagnosis of ZIKV.

ZIKV infection is diagnosed in the laboratory by nucleic acid amplification tests (NAATs) to detect viral RNA^20–24^ or by ELISA to detect IgM or IgG antibodies^21,25^. The NAATs such as RT-PCR and other technologies (e.g. recombinase polymerase amplification) are highly accurate, and RT-PCR is considered the gold standard to confirm ZIKV infection^21,24^. RT-PCR, however, requires a step for viral RNA extraction prior to the assay and the use of expensive equipment, such as thermal cycler, to conduct the test. Moreover, there is a risk of reduced sample quality due to RNA degradation during transportation to the laboratory. For ELISA, serological cross-reaction between ZIKV and other circulating flaviviruses like DENV makes accurate diagnosis with serology difficult^21,26^.Therefore, novel diagnostic technologies that can be conducted at the point-of-care or in regional laboratories are greatly needed to control ZIKV infections.

Reverse transcription loop-mediated isothermal amplification (RT-LAMP) is a rapid, sensitive RNA detection method performed under isothermal conditions using four or six unique oligonucleotide primers^27,28^. Since LAMP reactions can be performed with simple inexpensive equipment, RT-LAMP assays can be conducted in the field and by under-funded laboratories^29^. We previously developed a RT-LAMP assay using a portable isothermal amplification and detection device for Ebola virus in response to the recent outbreak of Ebola virus disease in West Africa, and the assay has been deployed for field surveillance in Guinea^30,31^. Here, we developed a RT-LAMP assay for the detection of ZIKV with a portable battery-powered device. Then, we evaluated the utility of this assay for molecular diagnosis using clinical specimens collected from the recent ZIKV outbreak in Brazil.

## Results

### Sensitivity

**Figure 1:**
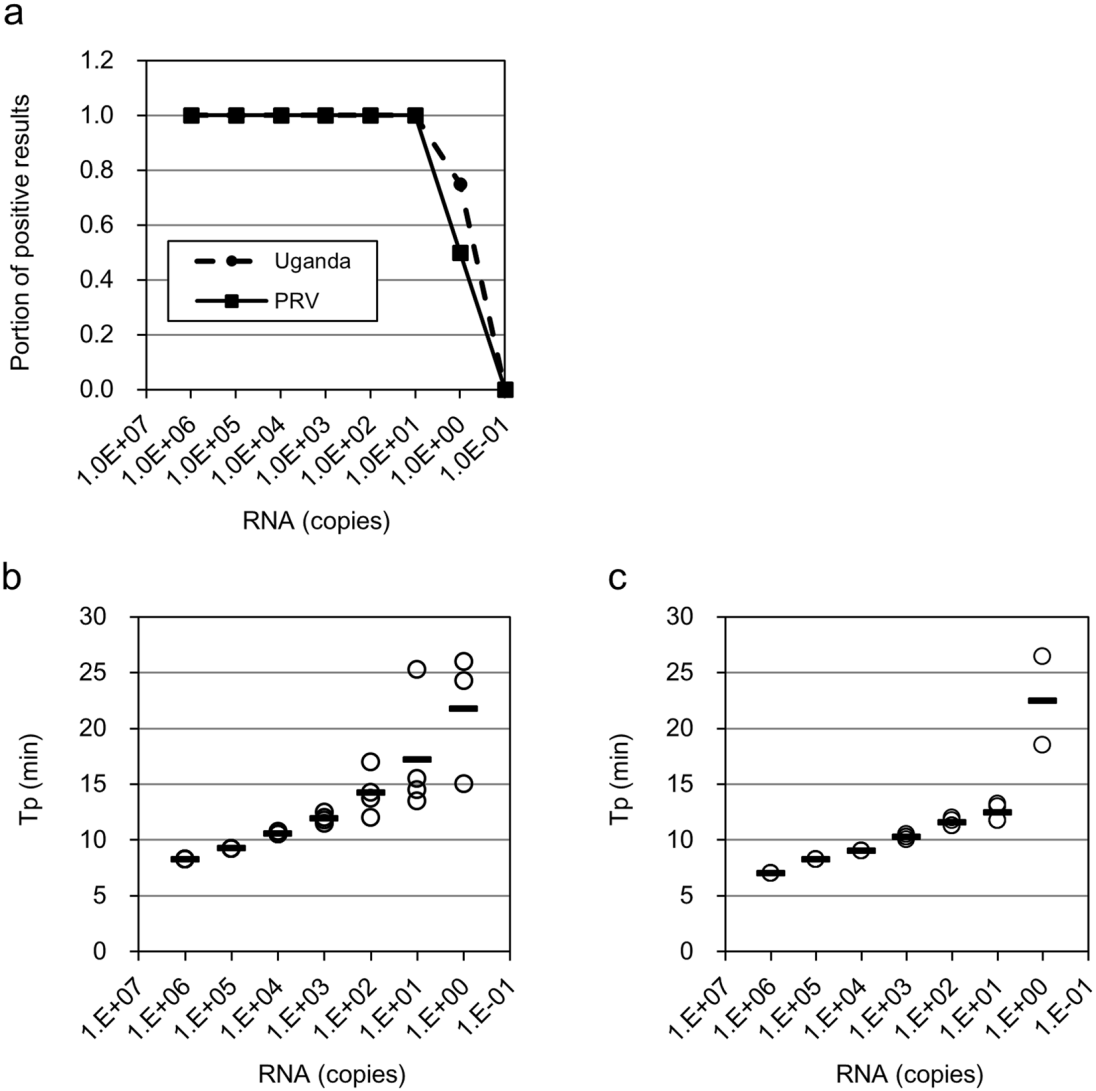
Sensitivity and detection time of the RT-LAMP assay for Zika virus (ZIKV). (**a**) Ten-fold serial dilutions of the RNA standards (976Uganda and PRVABC59 strains) were detected by the RT-LAMP assay. All reactions were performed in quadruplicate. Time to obtain positive results (Tp) for reactions with 976Uganda (**b**) and PRVABC59 (**c**) were determined using Genelyzer FIII. Each circle indicates the Tp for each reaction. Bars are the mean times of detection at the indicated dilutions.

We designed ZIKV genotype-specific LAMP primers that targeted conserved sequences in the E protein-coding region (Table 1). Each genotype-specific primer recognised the same genomic position. To detect all known ZIKV strains, we used a mixture of primers specific for each genotype in a single reaction. First, we examined the sensitivity of the assay using serial 10-fold dilutions of *in vitro* synthesised standard RNAs from strain 976Uganda, which was isolated from a rhesus macaque in Uganda, and strain PRVABC59, which was isolated at the Centres for Disease Control and Prevention (CDC) from a patient who travelled to Puerto Rico in 2015^32^. Ten copies of the RNA standards were detected from both strains in quadruplicate reactions (Fig. 1a). The times to obtain positive results (Tp) for RNA standards ranging from 10^6^ to 10^1^ copies were mostly less than 15 min, and within this range, Tp was correlated with the number of RNA copies (Fig. 1b and c). Single copies of the standard RNAs from the 976Uganda and PRVABC59 strains were detected with 75% and 50% positivity, respectively, and Tp values were dispersed. These results suggested that the RT-LAMP assay could be used as a rapid, sensitive diagnostic test for ZIKV, the Tp value (i.e., less than 15 min) can be used as an indicator of the number of RNA copies in each reaction.

**Table 1:** Sequences of LAMP primers.

Name	Type	Position*	Sequence (5ʹ–3ʹ)^†^	Specificity
ZIK-As4-F3	F3	1053–1071	AACATGGAGGTTGTGTCAC	Asian genotype
ZIK-As4-B3	B3	1315–1332	AACTTAGCGCATGTCACC	
ZIK-As4-FIP	FIP (F1c + F2)	1174–1192, 1114–1133	GCTGTCCGAAGCCATGTCT-TACAACAACAGTCAGCAACA	
ZIK-As4-BIP	BIP (B1c + B2)	1199–1220, 1262–1279	CCAACACAAGGTGAAGCCTACC-CCAGCCTCTGTCCACTAA	
ZIK-As4-LF	LF	1149–1170	ATTGATGCCTCATAGCAGTAGG	
ZIK-As4-LB	LB	1222–1242	TGACAAGCAATCAGACACTCA	Asian & African genotype
ZIK-Af41-F3	F3	1053–1071	AACATGGAGGTTGCGTCAC	African genotype
ZIK-Af41-B3	B3	1315–1332	AACTTGGCACATGTCACC	
ZIK-Af41-FIP	FIP (F1c + F2)	1174–1192, 1114–1133	ACTGTCCGAAGCCATGTCC-CACGACAACGGTTAGTAACA	
ZIK-Af41-BIP	BIP (B1c + B2)	1199–1220, 1262–1279	CCAACACAAGGTGAAGCCTACC-CCAACCTCTGTCCACCAA	
ZIK-Af41-LF	LF	1149–1170	ATTGATGCCTCGTAGCAATAGG	

*Primer position in ZIKV strain MR766 (accession number: NC_012532). ^†^Underlining indicates the positions of nucleic acids adapted to the African genotype.

### Specificity

**Figure 2:**
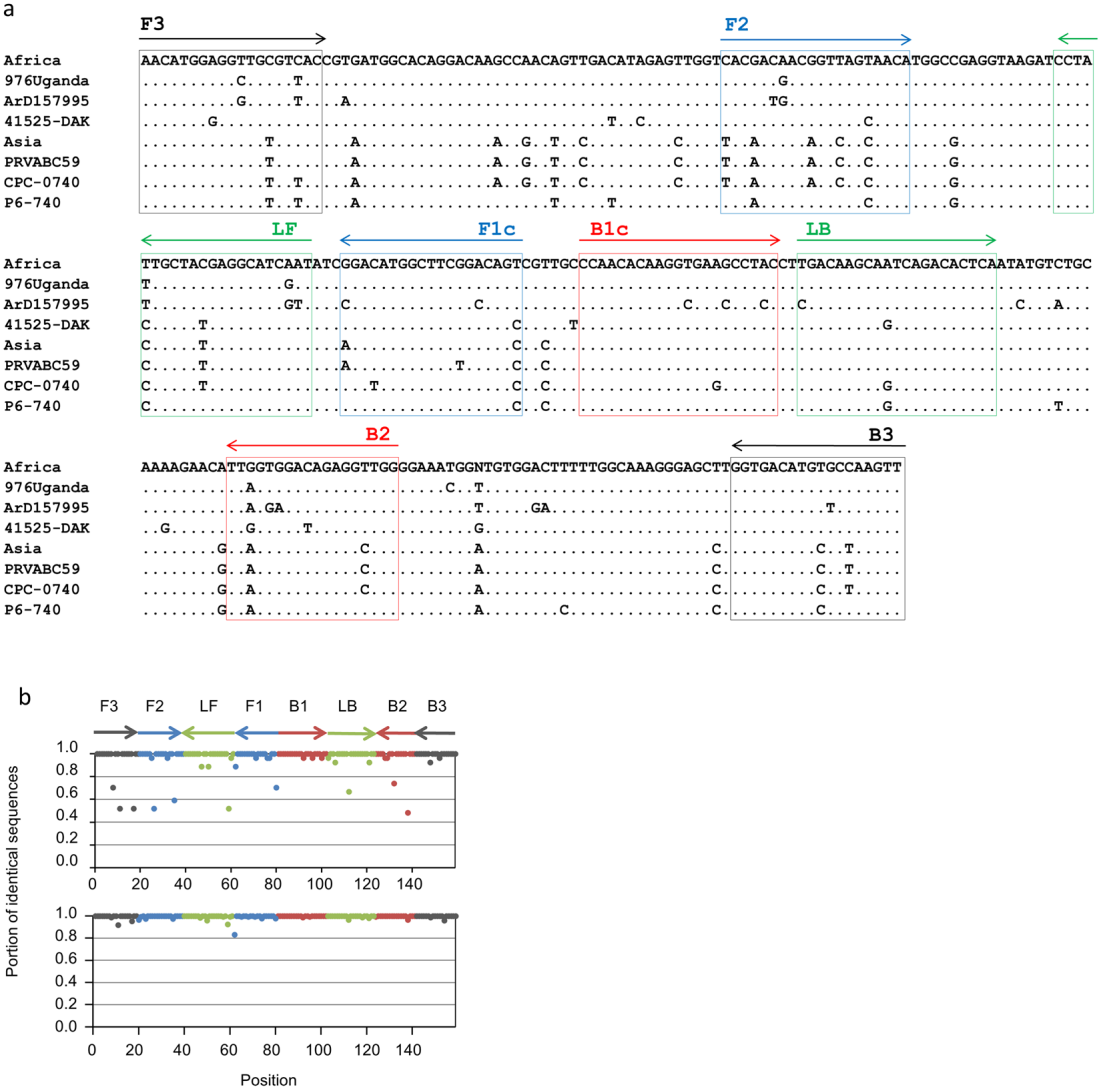
Specificity of the LAMP primers for ZIKV sequences. Alignment of ZIKV sequences and positions of LAMP primers (**a**). Boxes are the sites recognised by each oligonucleotide primer and arrows show the direction of each primer. Africa and Asia in the alignments indicate the consensus sequences of African and Asian genotypes, respectively. The accession numbers for the strains are LC002520.1, KF383118, KF955591, KU501215, HQ234499, and KU681082 (from top to bottom). Proportion of African (upper) and Asian (lower) genotype sequences that had identical nucleic acids with primers at respective positions in the LAMP primers (**b**).

We evaluated the specificity of each primer *in silico* using 176 ZIKV strain sequences available in GenBank as of November 2016. Twenty-seven sequences of African genotype isolates collected in 1947–2001 and 149 sequences of Asian genotype isolates collected in Southeast Asia and Polynesia in 1966–2014 as well as from the current outbreak in the Americas were used for this analysis. The LAMP primers consist of 159 nucleotides in total length and recognise eight separate sites on ZIKV genome (Fig. 2a). We determined the proportion of sequences that had identical nucleotides at each position for either the Asian or African genotype primers (Fig. 2b). African genotype ZIKV sequences had identical residues at 129 out of 159 positions (81.1%) in the African or Asian genotype-specific primers. At 14 positions in the primer recognition sites, more than 10% of African genotype ZIKV sequences had mismatched nucleotides. At six positions, scattered in the F3, F2, LF, and B2 sites in the Asian and African genotype primers, more than 40% of the African genotype ZIKV sequences had nucleotide differences (Fig. 2b, upper panel). Asian genotype sequences showed greater identity than African genotype sequences to the LAMP primers. Asian genotype sequences had identical residues at 131 out of 159 positions (82.4%) in the Asian or African genotype primers. For one residue at the 3′ terminus of the F1 site of the FIP primers, 16.8% of the Asian genotype sequences had nucleotide differences (Fig. 2b, lower panel). To assess the primer specificity for ZIKV strains, we synthesised RNAs with the partial genome sequences of two African genotype ZIKV strains, 41525-DAK and ArD157995, and two Asian genotype ZIKV strains, P6-740 and CPC-0740, which had more mismatched nucleotides against the primer sequences compared with the average for all strains. These RNA sequences were also detected using the RT-LAMP assay, in addition to the sequences in the 976Uganda and PRVABC59 strains (Table 2). Furthermore, no cross-reactions with other tested arboviruses, including DENV, YFV, West Nile virus (WNV), CHIKV, and Rift Valley fever virus (RVFV), and *Plasmodium falciparum* were observed. These results suggested that the RT-LAMP assay developed here was highly specific for detecting ZIKV strains of both African and Asian genotypes.

**Table 2:** Species specificity of ZIKV RT-LAMP.

Family	Genus	Species	Strain	Amount of RNA/DNA	Results of RT-LAMP
*Flaviviridae*	*Flavivirus*	*Zika virus*	976Uganda	2.0 × 10^2^ copies	+
		*Zika virus*	PRABC59	2.0 × 10^2^ copies	+
		*Zika virus*	ArD157995^*^	2.0 × 10^2^ copies	+
		*Zika virus*	41525-DAK^*^	2.0 × 10^2^ copies	+
		*Zika virus*	CPC-0740^*^	2.0 × 10^2^ copies	+
		*Zika virus*	P6-740^*^	2.0 × 10^2^ copies	+
		*Dengue virus serotype 1*	Hawaii	2.4 × 10^4^ copies	−
		*Dengue virus serotype 2*	ThNH7/93	9.5 × 10^4^ copies	−
		*Dengue virus serotype 3*	PhMH-J1-97	2.2 × 10^5^ copies	−
		*Dengue virus serotype 4*	SLMC 318	2.1 × 10^3^ copies	−
		*Yellow fever virus*	17D	2.7 × 10^4^ copies	−
		*West Nile virus*	NY99	3.5 × 10^4^ copies	−
*Togaviridae*	*Alphavirus*	*Chikungunya virus*	10Mdy30	1.7 × 10^5^ copies	−
*Bunyaviridae*	*Phlebovirus*	*Rift Valley fever virus*	SPU22/07	5.8 × 10^5^ copies	−
*Plasmodiidae*	*Plasmodium*	*Plasmodium falciparum*	3D5	0.5 ng	−

*Synthesised partial genomic RNA sequences were used for these strains.

### Detection of ZIKV in virus-spiked samples

In the routine molecular diagnosis of ZIKV infection, blood and/or urine is used, since viral RNA can be detected in these clinical specimens during the acute phase of infection. The feasibility of using the RT-LAMP assay for clinical specimens was evaluated using ZIKV-spiked human serum and urine samples. We prepared human serum and urine spiked with four-fold serially diluted ZIKV strain 976Uganda, and obtained samples with titres of 232.7–0.9 TCID_50_/mL. The sensitivity of the RT-LAMP assay was compared to that of the real time RT-PCR (rRT-PCR) assay developed by the CDC^21^. Using the RT-LAMP assay, we detected viral RNA in both serum and urine samples at a titre of 14.5 TCID_50_/mL in quadruplicate reactions. The Ct values in the rRT-PCR were 34.6 ± 0.8 and 35.4 ± 0.4 for serum and urine samples, respectively, which corresponded to 44.3 and 23.5 genome equivalents (geq) per reaction, respectively (Table 3). Using the rRT-PCR assay, we detected viruses in both serum and urine samples at a titre of 3.6 TCID_50_/mL, which corresponded to 8.8 and 8.9 geq per reaction, respectively; however, the RT-LAMP assay failed for these samples, suggesting that the RT-LAMP assay was less sensitive than the CDC rRT-PCR assay for ZIKV detection. Together with the results obtained using standard RNA shown in Fig. 1, the limit of detection of the RT-LAMP assay was estimated to be 10 copies per reaction. The assay may be sufficiently sensitive for detecting ZIKV in clinical specimens.

**Table 3:** Detection of ZIKV in virus-spiked urine and serum samples.

Sample	TCID_50_/mL	RT-LAMP		rRT-PCR		geq/test
		Positive	Tp (min)	Positive	Ct	
Serum	232.7	4/4	12.5 ± 0.5	4/4	31.4 ± 0.2	329.5
	58.2	4/4	13.4 ± 0.9	4/4	33.1 ± 0.4	109.7
	14.5	4/4	14.3 ± 1.5	4/4	34.6 ± 0.8	44.3
	3.6	0/4	—	4/4	37.0 ± 0.7	8.8
	0.9	0/4	—	0/4	—	—
	mock	0/4	—	0/4	—	—
Urine	232.7	4/4	12.3 ± 0.4	4/4	31.0 ± 0.2	423.0
	58.2	4/4	14.4 ± 0.4	4/4	33.1 ± 0.2	105.8
	14.5	4/4	14.6 ± 2.1	4/4	35.4 ± 0.4	23.5
	3.6	1/4	23.8	4/4	37.1 ± 0.8	8.9
	0.9	0/4	—	1/4	37.5	5.8
	mock	0/4	—	0/4	—	—

### Clinical evaluation of the RT-LAMP assay

We conducted a clinical evaluation of this assay using samples from patients with suspected arbovirus infection in the states of Paraíba and Pernambuco, Brazil in February–July 2016. The samples included 90 plasma/serum and 99 urine samples from 120 suspected arbovirus infection cases, including paired samples from 69 cases. To evaluate the diagnostic accuracy of this assay, we simultaneously conducted the CDC rRT-PCR assay as a reference test. In the RT-LAMP assay, eight out of sixteen serum samples collected in Pernambuco state in February 2016 were positive. However, all 74 plasma samples as well as urine samples collected in Paraíba state in March and July 2016 were negative. These results were concordant with those of the reference rRT-PCR assay (Table 4). The RT-LAMP assay did not show any false-positive results, even for six confirmed DENV samples (data not shown). The Ct values of these eight ZIKV-positive samples were 19.5–22.9, and the viral loads were estimated to be 1.6 × 10^6^–1.4 × 10^7^ geq/mL using the viral RNA standards (Table 5). These viral titres were higher than those reported in previous studies. To examine whether the assay can detect viral RNA in samples with lower titres, we randomly selected two ZIKV-positive samples confirmed in this study, MRL51 and MRL53, and conducted a dilution test (Table 6). While the RT-LAMP failed to detect samples with the Ct value > 37, however, it detected viral RNA at the Ct < 37, consistent with our earlier results obtained using the virus-spiked serum and urine samples (Table 3). These results show that the rRT-LAMP assay had sufficient specificity for the detection of ZIKV as a molecular diagnostic test. The assay can be used to detect an amount of viral RNA equivalent to that yielding Ct values of 36–37 in the reference rRT-PCR test.

**Table 4:** Detection of ZIKV in samples from patients with suspected arbovirus infection collected in Paraíba and Pernambuco, Brazil in 2016.

Period	State	Type	No. samples	RT-LAMP		rRT-PCR	
				Positive	Negative	Positive	Negative
February, 2016	Pernambuco	Serum	16	8	8	8	8
March, 2016	Paraíba	Plasma	65	0	65	0	65
		Urine	69	0	69	0	69
July, 2016	Paraíba	Plasma	9	0	9	0	9
		Urine	30	0	30	0	30
Total		Serum/Plasma	90	8	82	8	82
		Urine	99	0	99	0	99

**Table 5:** Viral load in ZIKV-positive samples tested in this study.

Sample ID	RT-LAMP (Tt, min)	rRT-PCR (Ct)	Virus load (geq/ml)
LAV01	8.5	20.4	7.9 × 10^6^
LAV04	8.8	20.8	6.2 × 10^6^
LAV08	7.8	19.5	1.4 × 10^7^
MRL51	8.3	21.6	3.7 × 10^6^
MRL53	9.0	22.9	1.6 × 10^6^
MRL55	8.5	21.4	4.2 × 10^6^
MRL56	8.8	21.3	4.5 × 10^6^
MRL57	8.8	21.2	4.8 × 10^6^

**Table 6:** Detection of ZIKV by RT-LAMP and rRT-PCR using diluted ZIKV-confirmed samples.

ID	Dilution (×10^2^)	Estimated RNA copies	RT-LAMP		rRT-PCR	
			Positive	Tp (min)	Positive	Ct
MRL51	1	116.0	3/3	12.4 ± 1.0	3/3	32.9 ± 0.1
	3	38.7	3/3	12.8 ± 1.3	3/3	34.4 ± 0.1
	9	12.9	2/3	13.2 ± 1.1	3/3	36.3 ± 0.6
	27	4.3	0/3		2/3	36.7 ± 0.1
	81	1.4	0/3		1/3	37.2
	243	0.5	0/3		0/3	
MRL53	1	51.2	3/3	11.9 ± 0.4	3/3	34.5 ± 0.1
	3	17.1	3/3	15.8 ± 2.8	3/3	36.2 ± 0.2
	9	5.7	0/3		2/3	37.8 ± 0.1
	27	1.9	0/3		0/3	
	81	0.6	0/3		0/3	
	243	0.2	0/3		0/3	
No template control			0/3		0/3	

.

## Discussion

We developed a rapid molecular detection assay for ZIKV in response to the recent outbreak in South America. LAMP assays and modified diagnostic methods for ZIKV have been reported; however, these molecular techniques have never been evaluated for clinical use^33–36^. This is the first evaluation of the clinical usage of a LAMP assay for molecular diagnostic testing in the recent outbreak of ZIKV infections. Since ZIKV shares a vector with DENV and CHIKV, these viral diseases can occur simultaneously, and Northeast Brazil is an endemic area for Dengue and Chikungunya^37^. Numerous severe mosquito-borne diseases, including arbovirus infections as well as Malaria, share clinical symptoms during the acute phase. However, ZIKV infection is generally associated with mild symptoms. A major concern with respect to molecular diagnostic testing for ZIKV is the potential for cross-reactivity with other flaviviruses, especially DNEV, which have close antigenic relation with ZIKV^21,23,25,38^. In contrast, our assay showed no cross-reactions with other arboviruses or *P. falciparum*, and did not show false-positive results when applied to ZIKV-negative samples. These results indicated that the RT-LAMP assay is specific for the detection of ZIKV and is a reliable molecular diagnostic test.

Another potential limitation of molecular diagnostic testing is that ZIKV-infected samples often have low titres after the acute or early phase of infection due to rapid clearance by the host immune system. This makes it difficult to identify ZIKV cases, even using RT-PCR-based tests. The limit of detection for this assay was 10 copies for both genotypes. The assay was slightly less sensitive than the CDC rRT-PCR test, which was commonly used to confirm ZIKV infection during the recent outbreak. ZIKV-infected clinical samples often show high Ct values (>35)^19,23^. However, the ZIKV-positive samples detected in this evaluation showed Ct values of less than 22.9 (more than 1.6 × 10^6^ geq/mL), which was a higher titre than that reported in other studies. To confirm its clinical utility, this assay should be tested using samples with lower titres or borderline ZIKV infections.

It has been reported that viral RNA can be detected for longer periods in urine than in blood^19,23^. Therefore, we considered urine to be one of the best sample types for detecting ZIKV infections. Recently, Paz-Bailey *et al*. reported contradictory results for the persistence of viral RNA in blood samples of ZIKV patients; RNA can be detected 1 or 2 weeks after the onset of illness^17^. In some cases, viral RNA can also be detected at higher titres in saliva than in blood, but persists for shorter periods^15,17^. It is necessary to determine the sample types suitable for the RT-LAMP assay and to establish a standardised RNA extraction protocol adjusted to each clinical specimen type in order to improve the sensitivity of this assay.

Owing to the sequence diversity among ZIKV isolates, we designed LAMP primers specific for each genotype and used a mixture of these primers to detect all known isolates of both African and Asian genotypes. As shown in Fig. 2, we conducted an *in silico* evaluation of each primer using available ZIKV sequences. African genotype strains supposedly have a longer history of circulation in African mosquitos and humans than that of Asian genotype strains^11^, and African genotype sequences showed a lower identity at some positions in the LAMP primers. The LAMP primers designed here showed high identities at most positions against the sequences of strains involved in the recent outbreak on the American continent, as well as its ancestral Southeast Asian and Polynesian isolates. During the outbreak of ZIKV in Americas, confirmed or probable ZIKV-infected cases has been continuously reported in Southeast Asia^39^. Our assay will be useful for virus detection and may contribute to preparedness for future outbreaks in these ZIKV endemic countries as well as in Asia and Africa. However, the evolution of ZIKV sequences must be constantly monitored to guarantee primer specificity.

Using samples obtained from subjects with suspected arbovirus infection, we did not find any ZIKV-positive samples in Paraíba in March or July 2016 by rRT-PCR or our RT-LAMP test. These samples were collected from patients within 1 or 2 weeks after the onset of arbovirus infection-like symptoms as part of an education and follow-up campaign for cardiovascular diseases. Many samples might have been collected after the acute or early stage of infection. In addition, when this campaign was conducted, the prevalence of ZIKV infection may have been low, since most cases were reported from November 2015 to March 2016^40^, which is closely linked to the ecology of the vector *Aedes* mosquito.

The main advantages of this assay are its speed (positive results can be obtained within 15 min) and the use of a battery-operated portable device. Since the device has a user-friendly interface, training is not necessary to conduct the assay and interpret the results. Recently, freeze-dried reagents for LAMP assays have been made available, making cold-chain-free LAMP assays a possibility. Our assay is suitable for use in field surveillance or remote areas where it is difficult to implement laboratory diagnostic tests. The assay should be evaluated in a prospective study to confirm its utility for molecular diagnostic testing, especially under limited resources and by field laboratories in ZIKV endemic countries.

In this paper, we successfully developed a RT-LAMP assay for the detection of ZIKV by designing Asian and African genotype-specific primers. The assay showed results consistent with those of the reference rRT-PCR assay in diagnostic tests with suspected cases of ZIKV infection. Our results provide a potential new molecular diagnostic test for ZIKV and may serve as a basis for the development of alternative rapid diagnostic techniques to prepare for potential outbreaks.

## Methods

### Cells and viruses

Vero 76 cells were obtained from the Health Science Research Resources Bank (JCRB9007) and were maintained in Dulbecco’s modified Eagle’s medium (DMEM) supplemented with 1% penicillin/streptomycin and 10% foetal bovine serum (FBS). ZIKV strain 976Uganda was kindly provided by Dr. Shigeru Tajima (National Institute of Infectious Diseases; NIID). The virus was propagated in Vero 76 cells grown in DMEM supplemented with 2% FBS. Two days after infection, culture supernatants were harvested, clarified by low-speed centrifugation, and then stored as virus stock at −80 °C until use. The infectious titre of the virus stock was determined by the 50% tissue culture infective dose (TCID_50_) using Vero 76 cells; titres are expressed as TCID_50_/mL. Viral RNA was extracted from 140 μL of infected culture supernatant using the QIAamp Viral RNA Mini Kit according to the manufacturer’s protocol. The RNA was eluted in 60 μL of elution buffer and stored at −80 °C until use. Viral RNA from ZIKV strain PRABC59 was kindly provided by Dr. Shigeru Tajima (NIID). Viral RNAs from other arboviruses, including DENV serotype 1–4, YFV, WNV, CHIKV, and RVFV, as well as genomic DNA from *P.falciparum* strain 3D7 were kindly provided by Dr. Kouichi Morita and Dr. Osamu Kaneko (Institute of Tropical Medicine, Nagasaki University).

### Preparation of RNA standards

RNA standards, consisting of partial genome sequences of ZIKV strains 976Uganda and PRVABC59, were amplified by RT-PCR using forward (5′-GGAGTCAGGATGGTACTTGTACC-3′) and reverse (5′-AAAATTGGATATTCAGGAACC-3′) primers with the PrimeScriptII High Fidelity One Step RT-PCR Kit (Takara Bio, Shiga, Japan). The reactions were performed using the TaKaRa PCR Thermal Cycler Dice with the following program: 45 °C for 10 min, 94 °C for 2 min, followed by 40 cycles of 98 °C for 10 s, 55 °C for 15 s, and 68 °C for 20 s. Amplified PCR fragments were cloned into the pCR2.1 vector using the TOPO-TA-Cloning Kit (Invitrogen, Carlsbad, CA, USA). The plasmids were digested with *Bam*HI, purified from the agarose gel slice using a column purification kit (Qiagen, Hilden, Germany), and used as templates for RNA synthesis. The partial genomic RNAs of each ZIKV strain were synthesised *in vitro* using T7 RNA polymerase (Promega, Madison, WI, USA) and purified using the RNeasy Mini Kit (Qiagen). The RNA concentration was determined by measuring the optical density at 260 nm (OD_260_) with a NanoDrop 2000 (Thermo Fisher Scientific, Waltham, MA, USA), and the RNAs were diluted in DEPC-treated water to achieve the desired concentrations.

### Primer design

LAMP primers for ZIKV detection were designed based on the coding sequences for the E protein. The ZIKV sequences available in GenBank were aligned using CLUSTALX to identify conserved regions. A consensus sequence for a region in the *E* gene was used to design LAMP primers using LAMP Designer (Optigene; http://www.optigene.co.uk/lamp-designer/). Primers specific for Asian genotype viruses were designed first, and then African genotype-specific primers were designed by adapting each position to the African genotype consensus sequence. The RT-LAMP assay required a set of six primers, two outer primers (F3 and B3), a forward inner primer (FIP), a reverse inner primer (BIP), a forward loop primer (LF), and a reverse loop primer (LB). The FIP consisted of the F1c sequence, which was complementary to the F1 and F2 sequences. The BIP consisted of the B1c sequence, which was complementary to the B1 and B2 sequences^27^. The LB primer was designed to detect both Asian and African genotype sequences. The sequences and locations of the oligonucleotide primers are shown in Table 1.

### RT-LAMP

RT-LAMP was performed with Isothermal Master Mix reagent (Optigene, West Sussex, UK) using the Genelyzer FIII real-time fluorescence detection platform (TOSHIBA Medical Systems, Otawara, Japan). The reaction mixture (total volume, 25 µL) contained 15 µL of Isothermal Master Mix; 1 µL of WarmStart RTx reverse transcriptase (1 U; New England BioLabs, Ipswich, MA, USA); 4 µL of the LAMP primer mix consisting of 5 pmol F3 and B3, 20 pmol FIP and BIP, 10 pmol LF and LB; and 5 µL of RNA sample (template). The assay was carried out using a mixture of primers specific for the Asian and African genotypes. All primers were cartridge-purified oligonucleotides purchased from Hokkaido System Science (Sapporo, Japan). The reaction was performed at 65 °C for 30 min, followed by a dissociation analysis at 95 °C–80 °C. DEPC-treated distilled water and RNA synthesised from 976Uganda or PRVABC59 were used for the negative and positive controls, respectively. Nonspecific amplification was excluded by comparing the melting temperature to that of the positive control^31^.

### Real time RT-PCR

Real time RT-PCR for ZIKV was performed using the QuantiTect Probe RT-PCR Kit (Qiagen) as reported previously^21^. The reaction mixture (total volume, 25 µL) contained 12.5 µL of 2× QuantiTect Probe RT-PCR Master Mix, 0.5 µL of QuantiTect RT Mix, 10 pmol each of primers 1086 and 1162c, and 5 pmol FAM-labelled 1086 probe for ZIKV. Then, aliquots of the RNA samples (2 µL) were added to the 25-µL reaction mixtures. Each reaction was performed using the 7500 Real-Time PCR System (Applied Biosystems, Tokyo, Japan) with a thermal cycle profile consisting of 48 °C for 30 min, 95 °C for 15 min, followed by 40 cycles of 95 °C for 15 s and 60 °C for 1 min. Cut-off values were set at Ct 38.5. To quantify viral RNA, a standard curve, generated with 10-fold serial dilutions of synthesised standard RNA from 976Uganda or PRVABC59, was used.

### Droplet digital PCR

Each arbovirus RNA listed in Table 2 except ZIKV was quantified by droplet digital PCR (ddPCR). The complementary DNA (cDNA) of each arbovirus RNA was synthesised from an extracted RNA stock using the SuperScript III First-Strand Synthesis System (Invitrogen) with forward primer for RVFV and reverse primers for DENV, WNV, YFV, and CHIKV, respectively (Supplementary Table 1). The primers used for ddPCR were designed using Primer3 (Supplementary Table 1). All 20-μL ddPCR mixtures contained 2× EvaGreen ddPCR Supermix (Bio-Rad, Hercules, CA, USA), 0.1 μM forward and reverse primers, and 2 μL of cDNA. Each oil compartment of the droplet generator DG8 cartridge (Bio-Rad) was filled with 70 μL of droplet generation oil for EvaGreen (Bio-Rad), and approximately 20,000 droplets were generated in each well by the QX200 Droplet Generator (Bio-Rad). The reactions were performed in a 40-μL droplet emulsion using a GeneAmp PCR System 9700 (Applied Biosystems) under the following thermal cycling conditions: 95 °C for 10 min, followed by 45 cycles of 94 °C for 30 s and 60 °C for 2 min, with a final step at 98 °C for 10 min. Controls without the template were used to monitor for signals from contamination or primer-dimer formation. The cycled droplets were read individually using the QX200 droplet reader (Bio-Rad) and analysed with QuantaSoft Droplet Reader software (Bio-Rad).

### Clinical specimens

Peripheral blood and urine samples were obtained from patients between 2 and 65 year old with suspected arbovirus infection, who presented with fever, rash, and/or arthralgia symptoms. Venous whole blood samples were collected in one VACUETTE^®^ Z Serum Separator Clot Activator and two Vacuette® EDTA Tubes (Greiner Bio-One, Kremsmünster, Austria). To one EDTA tube, RNA*later* (Thermo Fisher Scientific) was added at half the volume of the collected blood samples to prevent RNA degradation during transport. In total, 90 plasma/serum and 99 urine samples from 120 patients with suspected arbovirus infection, including paired samples from 69 cases, were used in this study. The separated plasma or serum samples and urine samples were stored at −80 °C until use. RNAs were extracted from sera and urine using the QIAamp Viral RNA Mini Kit (Qiagen) according to the manufacturer’s instructions. RNA samples were eluted with 60 µL of elution buffer and stored at −80 °C until use.

### Ethical declaration

This study was approved by the CCS-UFPE Ethical Committee (CAAE: 61603316.7.0000.5208) and all patients gave informed consent. Whole blood and urine samples were collected as part of an education and follow-up campaign for arboviruses and cardiovascular diseases conducted by LIKA in the states of Paraíba and Pernambuco, Brazil in February–July 2016. All experiments were performed in accordance with relevant guidelines and regulations.

## Electronic supplementary material

Supplementary Table 1
