# Supplementary material for: Development and evaluation of a rapid molecular diagnostic test for Zika virus infection by reverse transcription loop-mediated isothermal amplification
Source: Sci Rep. 2017 Oct 18;7:13503. doi: 10.1038/s41598-017-13836-9 (PMC5647432; doi:10.1038/s41598-017-13836-9)
Supplement: Supplementary file 1 — Supplementary Table 1 [file 41598_2017_13836_MOESM1_ESM.docx]

**Supplementary Information**

Development and evaluation of a rapid molecular diagnostic test for Zika virus infection by reverse transcription loop-mediated isothermal amplification

Yohei Kurosaki^1^, Danyelly Bruneska Gondim Martins^2^, Mayuko Kimura^1^, Andriu dos Santos Catena^2^, Maria Amélia Carlos Souto Maior Borba^2^, Sandra da Silva Matos^2^, Haruka Abe^1^, Rokusuke Yoshikawa^1^, José Luiz de Lima Filho^2^, Jiro Yasuda^1, 3^*

^1^Institute of Tropical Medicine (NEKKEN), Nagasaki University, Nagasaki 852-8523, Japan

^2^Laboratory of Immunopathology Keizo Asami (LIKA), Federal University of Pernambuco (UFPE), Recife 50670-901, Brazil

^3^Graduate School of Biomedical Sciences and Program for Nurturing Global Leaders in Tropical and Emerging Communicable Diseases, Nagasaki University, Nagasaki 852-8523, Japan

Table 1. Sequences of primers used for digital droplet PCR.

| Virus | Primer | Sequence (5ʹ–3ʹ) | Reference |
| --- | --- | --- | --- |
| *DENV serotype 1* | CDC DENV-1-F | CAAAAGGAAGTCGYGCAATA | [1] |
|  | CDC DENV-1-R | CTGAGTGAATTCTCTCTGCTRAAC |  |
| *DENV serotype 2* | CDC DENV-2-F | CAGGCTATGGCACYGTCACGAT | [1] |
|  | CDC DENV-2-R | CCATYTGCAGCARCACCATCTC |  |
| *DENV serotype 3* | CDC DENV-3-F | GGACTRGACACACGCACCCA | [1] |
|  | CDC DENV-3-R | CATGTCTCTACCTTCTCGACTTGYCT |  |
| *DENV serotype 4* | CDC DENV-4-F | TTGTCCTAATGATGCTRGTCG | [1] |
|  | CDC DENV-4-R | TCCACCYGAGACTCCTTCCA |  |
| *WNV* | WNV_10776_Fw | TGGGTTAACAAAGGCAAAMCA | This paper |
|  | WNV_10999_Rv | ATCCCAGGTGTCAATATGCTG |  |
| *YFV* | YFV_14_5UTR_Fw | GTGCTAATTGAGGTGCATTGG | This paper |
|  | YFV_105_5UTR_Rv | TCTGCTAATCGCTCAACGAAC |  |
| *CHIKV* | CHIKV_24_CDS_Fw | GCCTACCAGTTTCTTACTGCTC | This paper |
|  | CHIKV_161_CDS_Rv | CCTCAAACATGGGGTACG |  |
| *RVFV*^a^ | RVFV_S_70_CDS_Fw | AGAGTGGTCGTCGTGTTGTG | This paper |
|  | RVFV_S_180_CDS_Rv | TAGGACGATGGTGCATGAGA |  |

^a^Primers for RVFV were designed against the S-segment of its genome.

Reference

1. Santiago, G. A. et al. Analytical and clinical performance of the CDC real time RT-PCR assay for detection and typing of dengue virus. PLoS Negl Trop Dis **7**, e2311, (2013).
